# Retraction: The potency of lncRNA MALAT1/miR-155/CTLA4 axis in altering Th1/Th2 balance of asthma

**DOI:** 10.1042/BSR-2019-0397_RET

**Published:** 2023-06-05

**Keywords:** asthma, CTLA-4, lncRNA MALAT1, miR-155, T-bet/Gata3 ratio, Th1/Th2 balance

This article is being retracted from *Bioscience Reports* at the request of the Editor-in-Chief and the Editorial Board following receipt of a notification from a reader, alerting the Editorial Board to a duplicated row of bands in the Western blot of Figure 2F.

The authors were contacted regarding the concerns raised and provided the original Western blots for this image, however given that the backgrounds of original images differ significantly from those in the article file, concerns remain regarding the editing of this image. The Editorial Board stands by the decision to retract, the authors did not respond when asked to agree to the retraction.

